# Improving Therapeutic Ratio in Head and Neck Cancer with Adjuvant and Cisplatin-Based Treatments

**DOI:** 10.1155/2013/817279

**Published:** 2013-12-19

**Authors:** Loredana G. Marcu

**Affiliations:** ^1^School of Chemistry and Physics, University of Adelaide, Adelaide, SA 5000, Australia; ^2^Faculty of Science, University of Oradea, 410087 Oradea, Romania

## Abstract

Advanced head and neck cancers are difficult to manage despite the large treatment arsenal currently available. The multidisciplinary effort to increase disease-free survival and diminish normal tissue toxicity was rewarded with better locoregional control and sometimes fewer side effects. Nevertheless, locoregional recurrence is still one of the main reasons for treatment failure. Today, the standard of care in head and neck cancer management is represented by altered fractionation radiotherapy combined with platinum-based chemotherapy. Targeted therapies as well as chronotherapy were trialled with more or less success. The aim of the current work is to review the available techniques, which could contribute towards a higher therapeutic ratio in the treatment of advanced head and neck cancer patients.

## 1. Introduction

The major goal of cancer treatment is to improve the clinical outcome by increasing the therapeutic ratio (TR). Most commonly, the therapeutic ratio is quantitatively defined as the ratio between tumour control probability (TCP) and normal tissue complication probability (NTCP). In order to maximise the therapeutic ratio, tumour control needs to increase while normal tissue complications need to decrease. As with any other malignancy, the objective in the treatment of advanced head and neck cancer is to improve TR through both components: TCP and NTCP.

After decades of treatment optimisation via novel irradiation techniques, new cytotoxins and several adjuvant agents to improve tumour response to therapy, advanced unresectable head, and neck cancers are still a clinical challenge. Although the locoregional control showed improvement along the implementation of new treatment techniques, the death rate does not seem to decline for this malignancy [1].

Several randomised clinical trials showed a significant improvement in locoregional tumour control and disease-free survival when radiation was combined with cisplatin, as compared to radiation as a single agent [2, 3], reason why concurrent cisplatin-based chemoradiotherapy is nowadays the standard of care for advanced head and neck cancer patients.

Cisplatin is a platinum compound with complex properties when it comes to radiation-drug interaction. Through inhibition of DNA repair and cell cycle arrest cisplatin demonstrates radiosensitizing properties [4]. Furthermore, cisplatin exhibits radiosensitization of hypoxic cells due to scavenging of hydrated electrons by the platinum complex and formation of local concentrations of OH radicals, which eventually damage the DNA. Cisplatin was shown to have cytostatic properties by blocking the cells in the G2 phase of the mitotic cycle. It was demonstrated that cell cycle arrest at G2 is relevant to the *in vivo* action of cisplatin as subsequent lethal mitosis may be the most significant mechanism of cell death induced by this drug [5]. One of cisplatin's properties which is yet to be elucidated is the suppression of tumour neovascularization [6]. Yoshikawa et al. examined the effect of cisplatin on endothelial cell proliferation observing significant inhibition of endothelial cell growth for clinical drug concentrations. To date, the most important property of cisplatin as confirmed by preclinical and clinical studies is the ability to form DNA adducts. Cisplatin can form both intrastrand and interstrand adducts with the DNA [7]. Despite the low number of interstrand crosslinks (less than 1% of the total adducts) it was considered that these adducts are responsible for cisplatin's cytotoxic effect [8]. At the same time, there are studies relating cisplatin's cytotoxicity to the DNA-intrastrand crosslinks [9].

Irrespective of the exact mechanism that leads to radiosensitization cisplatin is a powerful drug, and since its clinical implementation it remains a fundamental cytotoxic agent for the management of head and neck cancer.

## 2. Challenges Imposed by Radiotherapy

Tumour hypoxia and accelerated proliferation of tumour cells during therapy (both radiotherapy and chemotherapy) remain some of the biggest challenges concerning the treatment of advanced head and neck cancer. The unpredictability of acute hypoxia in tumours often leads to treatment failure, and so does the rapid proliferation of tumour cells after the initiation of therapy. Cellular recruitment from the quiescent phase, accelerated accelerated stem cell division, abortive division, and loss of asynchronous stem cell division are thought to be the main mechanisms responsible for accelerated regrowth in squamous cell carcinomas of the head and neck [10–13]. Perhaps the most efficient method to overcome this burden is the alteration of standard fractionation in radiotherapy into hyperfractionated and/or accelerated radiotherapy. Several clinical trials confirmed the superiority of altered fractionation schedules in regard to tumour control as compared to standard (conventional) fractionation [14, 15].

Although altered fractionation increased locoregional control, there were trials that showed no treatment gain because of normal tissue complications. For instance, in the EORTC 22851 randomised trial [16], where hyperfractionated-accelerated radiotherapy was compared with the conventionally fractionated regimen, late toxicity nullified the gain in tumour control. The most common acute toxicities after radiotherapy reported in head and neck cancer patients are weight loss due to difficulties in swallowing, mucositis, xerostomia, and stomatitis, while dysphagia and late xerostomia are listed among late toxicities. There is, therefore, a price to pay for a better tumour control, though several times, normal tissue toxicity is a dose-limiting factor in both radiotherapy and combined chemoradiotherapy. The result is often treatment interruption, which further compromises tumour control.

## 3. Challenges Imposed by the Administration of Cisplatin

### 3.1. Normal Tissue Toxicity

While being one of the most potent chemotherapeutic agents, cisplatin is highly toxic to various organs. Nephrotoxicity and ototoxicity are some of the most commonly reported side effects during cisplatin-based chemotherapy. Other side effects, such as hematologic and central nervous system-related toxicities, are often dose-limiting factors or reason for treatment interruption. The rate of treatment completion is frequently reported to be below 100% due to adverse events. New radiotherapy delivery techniques might be a possible solution in reducing side effects due to better tumour conformity, as shown by studies comparing helical tomotherapy to conventional IMRT [17]. However, there is need for further studies to evaluate the real benefit of these techniques as the latest results show no significant difference among tomotherapy, IMRT, and 3D-CRT regarding normal tissue effects [18].

### 3.2. Risk of Second Primary Cancers

The risk of developing a second primary cancer after head and neck cancer treatment was shown to be strongly linked to the original risk factors, which initiated the first primary cancer, that is, smoking and alcohol consumption as well as the oncogenic human papillomavirus [19]. Thus the most common site for developing a second primary cancer in these patients is also the head and neck region.

**Figure 1:**
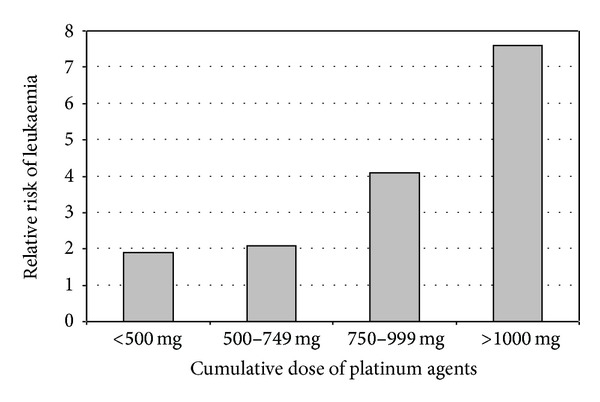
Risk of leukaemia as a function of the cumulative dose of platinum agents (based on Travis 1999 data [20]).

However, there is evidence that chemotherapy, particularly cisplatin, can induce carcinogenesis in patients treated with this agent for their primary malignancy. While proven to be toxic to normal tissue since the beginning of its clinical use, cisplatin was not shown to be carcinogenic until later. A large clinical study undertaken by Travis et al. showed an increased risk of leukaemia in patients previously treated with cisplatin [20, 21].  Figure 1 shows the increase in leukaemia risk together with the increase in cumulative dose of cisplatin. Although the data was based on a study undertaken on ovarian cancer patients treated with cisplatin, the findings may be applicable to head and neck cancer patients also [20].

## 4. Techniques to Maximise the Therapeutic Ratio in Head and Neck Cancer

The most commonly used methods to optimise the therapeutic ratio are reviewed below. While some of these techniques are widely accepted among the medical community (altered fractionation, cisplatin-based radiochemotherapy, and image-guided radiotherapy) others either are less successful in their clinical implementation or await more conclusive results (bioreductive drugs, normal tissue radioprotectors, and chronotherapy).

### 4.1. Combined Chemoradiotherapy

There is an extensive number of trials on head and neck cancer comparing the clinical effect of combined chemoradiotherapy versus radiotherapy alone. In order to collate and examine the results, a metaanalysis of the role of chemotherapy in head and neck cancer (MACH-NC) was published based on 93 randomised trials (conducted between 1965 and 2000) [22], which confirmed the superior outcome with combined chemoradiotherapy as compared to radiotherapy as a sole agent. While with concomitant chemotherapy a notable benefit was achieved, the results showed no clear justification for induction chemotherapy [22]. Still, the absolute benefit for chemotherapy after a 5-year follow-up period was only 4.5% (for concomitant chemotherapy the absolute benefit was 6.5%).

The advances in knowledge concerning head and neck radiobiology over the last few decades lead to clinical implementation of various altered fractionation schedules with and without chemotherapy. Altered fractionation radiotherapy combined with cisplatin-based chemotherapy became a common practice within the management of advanced head and neck cancer patients [23]. Intensity modulated techniques (IMRT) are employed for better tumour conformity either via hyperfractionated or accelerated radiotherapy concurrently with cisplatin-based chemotherapy.  Table 1 presents the most recent phases II and III clinical trial results employing intensity-modulated radiotherapy and cisplatin-based chemotherapy for advanced head and neck cancers. Both tumour control and normal tissue toxicity are presented, with variable results.

The studies presented in Table 1 have proven, once again, that combined chemoradiotherapy leads to increased locoregional tumour control when compared to radiation as a sole agent [24]. As also proven before, the outcome of nasopharyngeal cancer treatment tends to be better than that of other head and neck cancers [25]. Significantly improved treatment outcome was reported in the SAKK 10/94 trial in the combined arm (locoregional failure-free survival at 10 years: 40% versus 32%) [24]. 

The addition of bevacizumab [26] or cetuximab [27, 28] for an improved tumour control resulted in tolerable acute and late toxicity. Cetuximab-specific rash was reported by both trials.

Although locoregional control is significantly improved with the conversion of conventional therapy into IMRT, long-term survival remains at low rates, mainly due to tumour recurrence.

The mixed results achieved with altered fractionation combined with chemotherapy suggest that there is need for a careful selection of patients who would benefit from such therapy. The RTOG 0129 phase III randomised trial showed that combining cisplatin with altered fractionation (accelerated concomitant boost) does not give superior clinical results to standard fractionation combined with cisplatin [29]. The followup of 721 patients included in the RTOG 0129 trial presented no differences in the 5-year overall survival (59% versus 56%, *P* = 0.18), disease-free survival (45% versus 44%; *P* = 0.42), or locoregional failure (31% versus 28%; *P* = 0.76). Also, there was no significant difference in the overall grade 3-4 acute mucositis (33% versus 40%) or grade 3-4 late toxicity (26% versus 21%) [29]. Differences in radiobiological parameters, such as hypoxia and proliferation status might give indication on the patient group that would benefit from a more aggressive treatment.

One of the main challenges with injectable cisplatin is normal tissue toxicity. Therefore, the idea of oral administration of cisplatin was worth testing in order to investigate its level of tolerability. Tao et al. have designed a dose-escalation trial where oral cisplatin (CP Ethypharm) was administered in combination with radiotherapy to 18 head and neck cancer patients [30]. Four cisplatin dose levels were tested from 10 mg/m^2^/day to 25 mg/m^2^/day. Dose limiting toxicities in the form of gastrointestinal disorders were experienced for the highest dose hence the dose recommended for phase II trial was 20 mg/m^2^/day. Daily small doses of cisplatin have demonstrated good tolerability in the past even in injectable form [2]; therefore further studies are warranted to investigate the advantages of daily oral cisplatin in head and neck cancer patients.

A new platinum compound is on the horizon, namely, mitaplatin, which is a fusion between cisplatin and the orphan drug dichloroacetate previously developed to treat lactic acidosis [31, 32]. It was shown that mitaplatin elicits cytotoxic effects upon cisplatin-resistant head and neck tumour cells [31] by a dual killing mode: cisplatin interacts with the DNA while the action of the dichloroacetate is focused on the mitochondria by reversing the mitochondrial changes that confers cancer cells resistance to apoptosis. The ability of mitaplatin to selectively target cancer cells *in vitro* also with less normal tissue toxicity emerges as a strong foundation for further *in vivo* experiments.

### 4.2. Image-Guided Radiotherapy

Image-guidance represents today an important tool for increasing the therapeutic gain by better targeting the tumour, especially during fractionated radiotherapy when tumour shrinkage is expected over time and by better sparing of the surrounding normal tissue. Image-guided radiotherapy can be optimally achieved by employing cone beam CT during the course of radiotherapy then adapting the treatment plan according to the new tumour parameters [33].

Another imaging method, which assists in improving tumour control in head and neck cancer patients, is PET/CT. The functional properties supplied by PET together with the anatomical tumour delineation offered by CT provide a powerful tool in tumour classification and prediction of treatment outcome as well as selective targeting of hypoxic regions within the tumour. Beside 18F-FDG, which is still considered the standard radiotracer for PET imaging, there are hypoxia-specific radiotracers (F-MISO, F-FAZA) as well as proliferation-specific radiotracers (F-FLT) successfully used in clinical settings [34].

A clinical study conducted by Rothschild et al. [38] employing image-guided IMRT was undertaken on 131 patients with locally advanced pharyngeal carcinoma. The aim was to investigate the role of FDG-PET/CT guidance in predicting treatment outcome. One treatment arm comprised of 45 patients treated with PET/CT-based IMRT was control-matched with the second arm that included 86 patients treated with CT-based 3D conformal radiotherapy without image guidance. The 2-year overall survival for the PET/CT-IMRT arm was 91%, while in the control group only 54% of the patients were still alive 2 years after treatment. The significant increase in survival among the image-guided group shows the high potential of PET/CT in personalizing radiotherapy for head and neck cancer patients.

An innovative radiation therapy trial is currently accruing patients for analysing the predictive value of biological markers and 89Zr-cetuximab uptake in head and neck cancer treated with cisplatin versus cetuximab and standard radiotherapy versus redistributed radiotherapy. The focus of the ARTFORCE trial is on individualised treatment, using functional imaging for the assessment of dose escalation to the FDG-PET positive region and adaptive replanning accounting for anatomical changes during treatment [39].

### 4.3. Epidermal Growth Factor Receptor (EGFR) Inhibitors

The epidermal growth factor receptor (EGFR) plays a vital role in head and neck cancer development, growth, and metastatic spread and angiogenesis, owing to promotion of epidermal cell growth and regulation of cell proliferation. It was clinically proven that overexpression of EGFR leads to increased tumour proliferation and other growth-promoting behaviour. Of all head and neck squamous cell carcinomas a value as high as 90% exhibits overexpression of epidermal growth factor receptor [40].

A clinical trial conducted by Bonner et al. [41] aimed to investigate the efficacy of cetuximab when given concurrently with radiation as compared to radiotherapy alone. In the trial, 213 patients were randomised to radiotherapy alone and 211 patients to radiotherapy with cetuximab. The addition of cetuximab to radiotherapy significantly increased both 3-year locoregional control rates (47% versus 34%) and overall survival rates (55% versus 45%). The main cetuximab-related toxicities were acneiform rash and hypomagnesemia [41]. The group has also reported on the 5-year survival data while attempting to find a relationship between survival and the cetuximab-related rash [42]. Based on their clinical observations, patients enrolled in the cetuximab arm who developed grade 2+ cetuximab-induced rash had an improved overall survival compared to those patients who only presented with a mild rash.

The combined effect of cetuximab and cisplatin-based chemoradiotherapy was studied in a phase II trial for patients with advanced head and neck cancer [43]. Cetuximab was administered on a weekly basis concurrently with cisplatin (on weeks 1 and 4) and radiotherapy (with a boost dose for the last 2 weeks). Although the 3-year overall survival rate and locoregional control were 76% and 71%, respectively, the study was closed due to unexpected and significant adverse events.

Another anti-EGFR monoclonal antibody investigated as an agent for targeted therapies in head and neck cancer is panitumumab. As shown by preclinical studies, panitumumab has higher affinity for EGFR than other monoclonal antibodies developed to target EGFR [44]. This might explain the results of a phase I dose-escalation trial of panitumumab and paclitaxel combined with intensity modulated radiotherapy and carboplatin which indicated a high activity of the EGFR blocking antibody with well-tolerated side effects and an overall complete response rate of 95% [45]. Further studies to elucidate the long-term clinical effect of this agent are warranted.

### 4.4. Antiangiogenic Drugs

Angiogenesis inhibitors target those signalling molecules which stimulate the endothelial cells to migrate, divide, and form new blood capillaries. One such signalling molecule is the vascular endothelial growth factor (VEGF). Antiangiogenic drugs bind to VEGF before they could connect with the receptors of the endothelial cell to initiate the angiogenic process.

An angiogenic inhibitor that recognises and binds to VEGF is bevacizumab, a monoclonal antibody which was the first agent of its kind to slow tumor growth in glioblastoma, nonsmall cell lung cancer, and metastatic colorectal cancer patients [46]. Fury et al. [26] reported the results of a phase II clinical trial for advanced head and neck cancer that involved bevacizumab and cisplatin-based chemotherapy combined with intensity-modulated radiotherapy (see also Table 1). Progression-free survival rate at 2 years was 75.9% with an overall survival rate at 2 years of 88%. No increased toxicity with the addition of bevacizumab was reported.

An interesting study undertaken by Wang et al. [47] investigated the effect of bevacizumab, cetuximab, and cisplatin in various combinations on head and neck carcinoma in mice. Based on the results, the highest tumour control (expressed by the delay in tumour growth) and the maximum survival were achieved in a double-agent combination (bevacizumab-cisplatin) rather than the triple-agent combination of cetuximab-bevacizumab-cisplatin. The high apoptotic index in the triple-agent study as compared to the double-agent one (31.6 ± 12.0% versus 6.9 ± 1.3%) suggests that there might be an antagonistic effect of the monoclonal antibodies when administered together and also in combination with the platinum agent.

The same research group showed that bevacizumab has antitumour effect beyond antiangiogenesis, potentiating the cytotoxicity of cisplatin on squamous cell carcinomas [48]. The chemosensitizing property of bevacizumab was evidenced by *in vivo *studies via intratumoral injection. While the mechanism behind this behaviour is unclear, the results warrant further investigations.

### 4.5. Normal Tissue Radioprotectors

The rate and degree of normal tissue toxicity in head and neck cancer patients treated with radiation have justified the clinical need for a normal tissue radioprotector for reduction of side effects.

Amifostine is a commonly used normal tissue radioprotector, which has been trialled in combination with both radiotherapy and chemoradiotherapy. Amifostine was also proven to be an effective cytoprotector against the side effects caused by cisplatin [49]; therefore it was widely administered in cisplatin-based chemoradiotherapy for head and neck cancer [50]. Nevertheless, the results of clinical trials employing amifostine were inconclusive regarding the effect of this agent on the clinical outcome. To shed some light on the controversies raised by the use of amifostine, Bourhis et al. [51] analysed 12 trials encompassing 1119 patients (65% head and neck cancer patients) treated with radiotherapy ± chemotherapy. The conclusion of this metaanalysis was that amifostine did not have a negative impact on overall survival or progression-free survival.

The future use of amifostine remains uncertain owing to conflicting results regarding normal tissue toxicities. While some of amifostine's properties such as selectivity (i.e., lack of tumour protection) and safety were confirmed by several studies, the reduction in normal tissue toxicity was not always demonstrated [52].

### 4.6. Chronotherapy

It is a known and accepted fact that biological functions in humans are organised around a circadian (day/night) clock. Circadian rhythms are controlled by the suprachiasmatic nucleus of the hypothalamus, also known as the “master pacemaker”. Consequently, several biological processes are dictated by this clock including sleep, hormone secretion, and cell proliferation. Experimental findings suggest the existence of crosstalk between clock gene molecules and those molecules, which are responsible for cellular progression through the cell cycle [53].

Chronotherapy refers to treatment that is timed around this biological clock, which was shown to differ between normal and cancer cells [54, 55]. Chronotherapy could assist in reducing normal tissue toxicity in cancer patients if drugs pharmacokinetics and their target organs are known and understood.

One of the most common side effects during and after chemoradiotherapy of head and neck cancers are those involving the oral mucosa. The goal of chronotherapy is to take into account the biological clock of various tissues in trying to schedule treatment in the most opportune time for the tumour and the least harmful time for the normal cells. Therefore, investigating the peak times of oral mucosa cells could dictate the timing of treatment to diminish side effects. Bjarnason et al. have investigated the circadian variation of human oral epithelium through the expression of cell-cycle proteins [37] and verified the findings in a randomised clinical trial of 205 head and neck cancer patients [56]. The study concerning the circadian rhythm of the oral mucosa cells involved samples from healthy male volunteers and showed that cells in the S phase peaked around 3 pm, whereas cells in the M phase had their peak in the evening (Table 2).

Experimental studies support the evidence whereby the DNA synthesis rhythm in normal cells is in phase opposition with that of tumour cells, observation that is valid for the mitotic phase as well [54, 57]. Consequently, the most optimum treatment time for a high tumour cell kill would be in the morning, when most target cells are transitioning G_2_/M. During this time, normal cells would receive better protection, as they peak in G_1_.

There are clinical studies supporting the evidence whereby treatment timing according to cellular circadian rhythm can improve treatment outcome in head and neck cancer patients. Bjarnason et al. [56] conducted a randomised trial to verify the advantage of morning radiotherapy versus afternoon radiotherapy from a normal tissue perspective. They hypothesised that morning radiotherapy will lead to less normal tissue effects involving the oral mucosa, as oral epithelial cells peak in G_1_. Patients were randomised to morning (8–10 am) versus afternoon (4–6 pm) radiotherapy. The results showed a significant improvement in weight loss for the morning group as compared to patients receiving radiotherapy in the afternoon and reduced incidence of oral mucositis among male patients (49.4% versus 64.1%). It was suggested that a greater treatment time difference between the two arms could have lead to a greater advantage of morning radiotherapy.

It was shown that circadian dosing time could also influence drug-related toxicities [58]. Consequently, cisplatin should be less toxic to normal tissue if administered around 4 pm when the target organs (such as kidney and bone marrow) are less susceptible to cisplatin-caused damage. Positive results confirming the preclinical findings of platinum chronotherapy were reported by Focan et al. [59] in a study undertaken on oesophageal cancer patients. Normal tissue toxicity was considered excellent, with grade 3-4 oral mucositis occurring in only 11–23% patients, leucopenia in 6–19%, and thrombopenia in 18–50%.

The results achieved with chronotherapeutics in leukaemia patients, ovarian cancer patients, and metastatic colorectal cancer patients are even more pronounced. For instance, a twofold increase in survival and disease free rate after 5 years was reported in a chronotherapy trial involving children with acute lymphoblastic leukaemia when chemotherapy (antimetabolites) was administered in the evening (80% survival) versus morning treatment (40% survival) [60, 61]. The results indicate that malignant lymphoblasts are more susceptible to antimetabolites in the evening than in the morning hours.

To obtain conclusive results with head and neck cancer chronotherapy, there is need for further studies involving a multidisciplinary approach and an open-minded attitude towards less orthodox treatment methods that showed promising results in the past.

## 5. Conclusions

Advanced head and neck cancers are difficult to manage despite the large treatment arsenal currently available. The multidisciplinary effort to increase disease-free survival and diminish normal tissue toxicity is rewarded with better locoregional control and sometimes fewer side effects. Nevertheless, locoregional recurrence is still one of the main reasons for treatment failure.

In order to increase therapeutic ratio, there are methods to improve tumour control as well as normal tissue sparing. Some of the techniques to achieve these goals are listed below.

Techniques to increase TCP:

- optimum fractionation schedules;
- optimum timing between radiotherapy and cisplatin administration based on cisplatin's pharmacokinetics and pharmacodynamics as well as the interaction between cisplatin and radiation;
- knowledge of pretreatment radiobiological tumour parameters such as oxygenation status and cellular proliferative capacity;
- image-guidance during radiotherapy;
- EGFR inhibitors such as cetuximab;
- angiogenic inhibitors;
- chronotherapy involving knowledge of tumour circadian rhythm.

Techniques to decrease NTCP:

- optimum timing between radiotherapy and cisplatin administration;
- more conformal radiotherapy (IMRT);
- image-guidance during treatment;
- normal tissue radioprotectors such as amifostine;
- chronotherapy involving knowledge of normal tissue circadian rhythms, especially of the oral mucosa and bone marrow to diminish side effects of both radiotherapy and chemotherapy.

The latest treatment techniques combined with adjuvant and/or targeted therapies succeeded in increasing locoregional control in advanced head and neck cancer patients. The downside, however, is the increased rate of side effects. Furthermore, overall survival in this patient group has not seen any considerable improvement over the last decades. While there are promising results with targeted therapies involving monoclonal antibodies as well as with chronotherapy, the optimal treatment for advanced head and neck cancer patients is yet to be established.

## Figures and Tables

**Table 1:** Cisplatin-based chemoradiotherapy regimens employing IMRT techniques.

Clinical study	Patient no.	Radiotherapy	Chemotherapy	Clinical outcome
*Phase II trial * (Fury et al. 2012) [26] Bevacizumab with cisplatin plus intensity-modulated radiation therapy	42	IMRT total dose of 70 Gy	Cisplatin (50 mg/m^2^ on days 1, 2, 22, 23, 43, and 44) and bevacizumab (15 mg/kg on days 1, 22, and 43)	*TCP* progression-free survival rate at 2 years: 75.9%; overall survival rate at 2 years: 88% *NTCP*, no increased toxicity with the addition of bevacizumab.

*Phase III trial* (SAKK 10/94) (Ghadjar et al. 2012) [24] Concomitant cisplatin and hyperfractionated radiotherapy versus hyperfractionated radiotherapy alone	224	Median total dose of 74.4 Gy, 1.2 Gy twice daily 5 days per week	Two cycles of cisplatin (20 mg/m^2^ for 5 consecutive days during weeks 1 and 5)	*TCP* locoregional failure-free survival at 10 years: 40% versus 32%; metastasis-free survival: 56% versus 41%; cancer-specific survival at 10 years: 55% versus 43% in the combined arm versus radiotherapy alone *NTCP*, no difference in late toxicity.

*Phase II study* (Ma et al. 2012) [27] Concurrent cetuximab-cisplatin and intensity-modulated radiotherapy	30	IMRT median dose of 70 Gy	Cetuximab: initial dose of 400 mg/m^2^ 7–10 days before concurrent IMRT weekly cisplatin (30 mg/m^2^/week) and cetuximab (250 mg/m^2^/week)	*TCP* 2-year progression-free survival 86.5% *NTCP* grade 3-4 oropharyngeal mucositis: (87% and 33%) required short-term nasogastric feeding. Grade 3 radiotherapy-related dermatitis (20%) and 10% grade 3 cetuximab-related acneiform rash.

*Phase II trial* (Maguire et al. 2011) [35] Hyperfractionated intensity-modulated radiation therapy and concurrent weekly cisplatin	39	IMRT to high-risk planning target volume 70 Gy of 1.25 Gy twice daily fractions. Intermediate and low-risk PTVs of 60 Gy and 50 Gy, at 1.07 and 0.89 Gy/fraction	Cisplatin 33 mg/m^2^ weekly	*TCP* actuarial 3-year overall survival: 80%, progression-free survival: 82%, and locoregional control:87% *NTCP* grade 3 + toxicities mucositis (38%), fatigue (28%), dysphagia (28%), and leukopenia (26%).

*Clinical study* (Montejo et al. 2011) [28] Accelerated radiotherapy with concurrent chemotherapy	43	IMRT with simultaneous integrated boost (67.5, 60, and 54 Gy in 30 daily fractions of 2.25, 2, and 1.8 Gy)	Cisplatin 40 mg/m^2^ weekly or 100 mg/m^2^ every 3 weeks during radiotherapy + weekly cetuximab (3 patients only)	*TCP* complete response: 74.4%; estimated 5-year locoregional control: 82%; *NTCP *tolerable acute and late toxicities.

*Phase II trial* (Lu et al. 2010) [25] Weekly cisplatin with concurrent intensity-modulated radiation therapy	22	IMRT of 69-70 Gy at 2.12–2.3 per fraction delivered to the PTV (including nodes)	Cisplatin 40 mg/m^2^ weekly for six cycles	*TCP *1-year overall survival: 95.5%, local recurrence-free survival: 95.5%, regional recurrence-free survival: 100%, and distant metastasis-free survival: 100% *NTCP* mild to moderate. Grade 3 stomatitis (27%), all other toxicities (less than 10%).

*Clinical study* (Lee et al. 2007) [36] Concurrent chemotherapy and intensity-modulated radiotherapy	31	Median dose of 70 Gy at 2.12 Gy/fraction to the PTV_GTV_; 59.4 Gy at 1.8 Gy/fraction to the PTV of high-risk subclinical disease	Cisplatin 2 to 3 cycles 100 mg/m^2^ intravenously within 2 days every 3 weeks	*TCP* The 2-year local progression-free survival: 86%; regional progression-free survival: 94%; overall survival 63% *NTCP* grade 2 + mucositis occurred in 48% of patients; all had Grade 2 + pharyngitis during treatment.

**Table 2:** The circadian rhythm of normal oral mucosa cells (data from Bjarnason et al. [37]).

Cycle phase	Early G_1_	Late G_1_	S	G_2_	M
Peak time	6 am	11 am	3 pm	4 pm	9 pm
Phase marker	p27	p53	cyclin E	cyclin A	cyclin B
